# Identification and Validation of a Linear Protective Neutralizing Epitope in the β-Pore Domain of Alpha Toxin

**DOI:** 10.1371/journal.pone.0116882

**Published:** 2015-01-30

**Authors:** Jon Oscherwitz, Kemp B. Cease

**Affiliations:** 1 Division of Hematology-Oncology, Department of Internal Medicine, University of Michigan Medical School, Ann Arbor, Michigan, 48105, United States of America; 2 VA Ann Arbor Healthcare System, 2215 Fuller Road, Ann Arbor, Michigan, 48105, United States of America; National Institutes of Health, UNITED STATES

## Abstract

The plethora of virulence factors associated with *Staphylococcus aureus* make this bacterium an attractive candidate for a molecularly-designed epitope-focused vaccine. This approach, which necessitates the identification of neutralizing epitopes for incorporation into a vaccine construct, is being evaluated for pathogens where conventional approaches have failed to elicit protective humoral responses, like HIV-1 and malaria, but may also hold promise for pathogens like *S. aureus*, where the elicitation of humoral immunity against multiple virulence factors may be required for development of an effective vaccine. Among the virulence factors employed by *S. aureus*, animal model and epidemiological data suggest that alpha toxin, a multimeric β-pore forming toxin like protective antigen from *Bacillus anthracis*, is particularly critical, yet no candidate neutralizing epitopes have been delineated in alpha toxin to date. We have previously shown that a linear determinant in the 2β2-2β3 loop of the pore forming domain of *B. anthracis* protective antigen is a linear neutralizing epitope. Antibody against this site is highly potent for neutralizing anthrax lethal toxin in vitro and for protection of rabbits in vivo from virulent *B. anthracis*. We hypothesized that sequences in the β-pore of *S. aureus* alpha toxin that share structural and functional homology to β-pore sequences in protective antigen would contain a similarly critical neutralizing epitope. Using an in vivo mapping strategy employing peptide immunogens, an optimized in vitro toxin neutralization assay, and an in vivo dermonecrosis model, we have now confirmed the presence of this epitope in alpha toxin, termed the pore neutralizing determinant. Antibody specific for this determinant neutralizes alpha toxin in vitro, and is highly effective for mitigating dermonecrosis and bacterial growth in a mouse model of *S. aureus* USA300 skin infection. The delineation of this linear neutralizing determinant in alpha toxin could facilitate the development of an epitope-focused vaccine against *S. aureus*.

## Introduction

Identifying neutralizing determinants in critical pathogen molecules can inform the rational design of vaccines which are capable of eliciting precisely-focused immune responses. This epitope-focused vaccine approach is increasingly being evaluated for the development of vaccines for pathogens where conventional approaches have failed to elicit protective humoral responses, including the human immunodeficiency virus type-1, malaria and human respiratory syncytial virus, and typically requires identification of neutralizing determinants within the target molecules through epitope mapping of neutralizing antibodies, or prediction of such epitopes through structural modeling[1–5]. Conceptually, this approach may also have utility for the development of vaccines against pathogens, like *Staphylococcus aureus*, where the elicitation of humoral immunity against an array of virulence factors may ultimately be required for development of an effective vaccine[6–8]. Vaccination with an epitope-focused molecular vaccine comprised of a single molecule capable of eliciting functional antibody against multiple discrete epitopes on critical virulence factors could offer a number of advantages. These would include a simplified and less expensive path through pre-clinical development, including toxicology studies, and a likely reduction in the elicitation of antibody (Ab) which play no functional role in neutralizing or binding critical epitopes, as has been shown to occur with immunization with full length proteins in other models [9,10]. As in the use of a key subunit rather than a whole-killed vaccine, the reduced production of such extraneous Ab could decrease the potential for cross-reactivity of vaccinee sera with normal tissues which may also be a source of reactigenicity[11]. A poly-virulence factor, epitope-focused vaccine would employ an immunogenic platform capable of accomodating multiple epitope payloads while retaining excellent immunogenicity[12].

*S*. *aureus* produces dozens of molecules known to contribute to virulence, and each may represent a potential target for a *S*. *aureus* vaccine [13–17]. These include surface-expressed determinants including Protein A, IsdB, clumping factor A (ClfA) and capsular polysacharrides, and secreted exotoxins including alpha, beta and gamma hemolysin, the phenol-soluble modulins (PSMs), among others [18–20]. Alpha toxin (AT), or alpha hemolysin (Hla), encoded by the *hla* gene, is a prominent candidate among these factors, as evidence suggests that AT, a protein ubiquitously secreted by most strains of *S*. *aureus*, is a critical virulence factor in *S*. *aureus* infection, host interaction, and pathology [21–24]. Indeed, the high levels of virulence associated with community associated methicillin-resistant *S*. *aureus* (CA-MRSA) appears to be related in part, to the high levels of AT produced by these strains [18,25,26]. The primacy of AT as a virulence factor is also evident in the mouse models of *S*. *aureus* disease, where wild type methicillin-sensitive *S*. *aureus* (MSSA) and MRSA strains associated with human disease are highly virulent in the pneumonia, sepsis and dermonecrosis models, yet the respective *hla* deletion mutants are almost completely devoid of pathogenicity[27–29].

Studies in the early 1990s first demonstrated that passive immunization with rabbit antibody elicited to H35L, a non-toxic mutant of AT [30], is capable of mediating protection of mice from lethal *S*. *aureus* challenges, and more recently, active immunization of mice and rabbits with H35L or other detoxified forms of AT has been shown to confer significant protection in models of *S*. *aureus* pneumonia, sepsis and dermonecrosis [6,27,31–35]. Sub-unit vaccines have also proven efficacious in the mouse models, as two independent studies showed that immunogens comprised of sequences from the N-terminal segment of AT were capable of eliciting neutralizing antibody that mediated protection from challenges in the pneumonia and sepsis models[36,37]. Yet, despite the compelling data validating AT as a critical target of humoral immunity, there is a paucity of data on specific neutralizing epitopes within AT, especially with regard to linear neutralizing determinants that might be candidates for the design of an epitope-focused vaccine.

AT is a 293 residue protein that binds ADAM10 on the surface of host cells and then self-associates to form a heptameric structure that creates a pore in eukaryotic membranes [38,39]. The heptamer pore channel is a 14-stranded beta barrel formed by contributions of a beta-hairpin loop from each of the monomeric alpha-toxin molecules [40]. AT is a prototypical example of a multimeric pore-forming toxin, a category which also includes protective antigen (PA) of *B*. *anthracis*, and the pore forming domains of both toxins share structural and functional similarities as exemplified by a shared inhibition by β-cyclodextrin derivatives [41]. We have previously shown that a linear determinant in the 2β2–2β3 loop of the pore forming domain of PA is a potent neutralizing epitope. Immunization with linear and multiple antigenic peptide (MAP) immunogens or a recombinant protein targeting this sequence, referred to as the loop neutralizing determinant (LND), has been shown to elicit antibody capable of highly potent in vitro neutralization of lethal toxin, the two component toxin formed through interaction of PA and lethal factor. Importantly, LND-specific Ab was shown to mediate complete protection of rabbits from lethal aerosol challenge with *B*. *anthracis* Ames strain spores in vivo when such antibody responses were elicited by either MAPs or a recombinant protein [42–45]. Moreover, the protection in some animals was associated with relatively low levels of LND-specific Ab, potentially highlighting the vulnerability of sequences within the β-pore region of the PA molecule. The importance of using an epitope-focused vaccine for eliciting antibody specific for the LND in PA is revealed by the striking finding that immunization with whole PA does not elicit detectable quantities of antibody specific for the LND in rabbits, non-human primates and humans. Instead, elicitation of Ab against these relatively cryptic, yet vulnerable LND sequences in PA, requires immunization with an epitope-focused vaccine[44,45].

We hypothesized that sequence in the β-pore of alpha toxin (AT) that shares some homology with the LND sequence in PA, could contain a similarly critical neutralizing epitope. In an approach informed by available molecular data, we used an in vivo epitope mapping strategy employing peptide immunogens, in concert with an optimized in vitro alpha toxin neutralization assay (TNA) to establish the presence of this linear neutralizing epitope in AT, termed the pore neutralizing determinant (PND). We then employed the mouse dermonecrosis model of *S*. *aureus* disease to validate the PND as a potentially important target of protective humoral immunity.

## Materials and Methods

### Synthetic Peptides

MAPs and linear synthetic peptides used in this study were synthesized commercially (Bio-Synthesis, Inc., Lewisville TX). The synthesis of peptides in a branched chain configuration on a lysine backbone, now commonly referred to as MAPs, was first described by Tam et al. as a method for potentiating immune responses to peptide immunogens[46,47]. Linear synthetic peptides were HPLC purified to greater than 90% purity and included the peptides, a.a. 1–19 and a.a. 122–137 from alpha hemolysin (UniProtKB P09616). For all studies employing MAPs, 4-branch constructs were synthesized with the respective alpha toxin candidate B cell sequence positioned at the C-terminus, collinearly synthesized with the T* helper T cell epitope (EYLNKIQNSLSTEWSPCSVT) at the N-terminus as employed previously[44,48]. This epitope is a major histocompatibility locus (MHC) Class II-restricted CD4^+^ T cell epitope from the circumsporozoite protein of *Plasmodium falciparum* [49]. For some studies, rabbits were immunized with a mixture containing MAPS synthesized with T* helper epitope as well as separately with the P30 helper T cell epitope (FNNFTVSFWLRVPKVSASHLE), an MHC Class II restricted CD4+ T cell epitope from tetanus toxin [43,50]. Control MAPs used for this study consisted of 4-branch constructs with the T* and P30 helper epitopes separately linked to a B cell sequence (QSVEINCTRPNNNTRKSIHMGPGRAF) derived from the V3 loop of the envelope glycoprotein of HIV-1. Structural examination and comparison of 3-dimensional structures employed experimental and modeled structures available through the Protein Data Bank (rcsb.org) using the PyMOL Molecular Graphic System, version 1.6 (Schrödinger, LLC). Primary structure homology was studied using ClustalX version 2.1.

### Animals and Vaccinations

Approximately 6–8 week old female C57BL/6 and BALB/c mice (Charles River, Portage MI) were used for the mouse experiments. Passive immunization was performed as described in text with either rabbit antisera or affinity purified rabbit IgG. Rabbit IgG was purified from rabbit antisera on Protein A (Thermo Fisher Scientific Inc., Rockford IL) according the manufacturers protocol, sterile filtered, and adjusted to a concentration of approximately 1.0 mg/ml for injection. The neutralization ED_50_ of the purified Ab was 1,554. The concentration of PND-specific Ig within the Protein A purified sample was subsequently determined through affinity chromatography on 6% crosslinked beaded agarose to which the 119–139 peptide synthesized with an N-linked cysteine was covalently-immobilized. The column preparation and purification were performed according the manufacturers protocol (Sulfolink, ThermoFisher Scientific). The PND-specific antibody concentration in the affinity purified rabbit IgG used for the passive transfer studies was determined to be 0.690 mg/ml. For procurement of rabbit antisera, female New Zealand white (NZW) rabbits (Covance Research Products, Denver, PA) were immunized on day 0 with 250 μg of the respective MAP immunogens in an emulsion with CFA and were then boosted 3 times at two-week intervals with 125 μg of the respective MAP in an emulsion with IFA. For assessment of antibody responses, rabbits were bled 10–14 days after the final immunization. Mice and rabbits were cared for in accordance with the standards of the Association for Assessment and Accreditation of Laboratory Animal Care and all efforts were made to ameliorate animal suffering. At the conclusion of the studies, rabbits and mice were euthanized using barbiturate overdose or carbon dioxide asphyxiation, respectively. All animal procedures were approved by the Institutional Animal Care and Use Committee at the VA Ann Arbor Healthcare System (Permit Number-A3756–01), and Covance Research Products, (Permit Number-A3850–01).

### Bacterial Strains

*S*. *aureus* strain 8325–4, and the isogenic *hla*(-) deletion mutant are MSSA strains that have been described previously and were kindly provided by Dr. Timothy Foster (Trinity College, Dublin, Ireland)[51,52]. The MRSA strain LAC/USA300 was a generous gift of Dr. Michael Otto (NIH, Bethesda, MD). Strain SAP149 which was generously provided by Dr. Scott Stibitz (NIH), is a luminescent derivative of NRS384 and has been described elsewhere[32,53].

### Preparation of Bacteria for Dermonecrosis challenge model

For preparation of challenge stocks, *S*. *aureus* strains were grown overnight (ON) (Day 0) in trypic soy broth (TSB, Sigma Biochemicals, St. Louis, MO) at 37°C with shaking at 230 rpm in 200 ml Erlenmeyer flasks. For strain SAP149 only, cultures were supplemented with 10 μg/ml of chloramphenicol (Sigma Biochemicals). ON cultures were diluted 1:100 (Day 1) and were expanded for 3 hours at 37°C and 230 rpm until mid-exponential phase (approximately 0.7 OD at 600 nm). Approximately 30 mls of expanded culture was then spun down and pelleted at 3000 x g, washed twice with Dulbecco’s phosphate buffered saline (DPBS) and resuspended in 6 mls of DPBS. Optical density at 600 nm (1.0 OD unit = approximately 600 x 10^6^ CFUs *S*. *aureus*) was used to determine preliminary bacterial concentrations, and exact concentrations of bacterial inocula were determined from analysis of plate counts from serial dilutions of *S*. *aureus* on TSB agar plates.

### Dermonecrosis Model

On the day of challenge, mice were anesthetized with isoflurane, hair was removed from the back and hind quarters with electric clippers (Oster, blade #50) and shaver (Remington MicroScreen) and mice were then injected intradermally with 0.05 mls of *S*. *aureus* at a typical concentration ranging from approximately 400–600 x 10^6^/ml suspended in DPBS. Actual challenge doses were determined from bacterial plating and are indicated in the text or figure legends. Following inoculation, mice were followed for a period ranging from 2–6 days, at which time all mice were euthanized and dermonecrotic lesions were photographed. Chemical depilation was optionally performed as necessary to reveal the margins of lesions obscured by regrown or unshaven hair (VEET Fast Acting Gel Cream Hair Remover, Reckitt Benckiser, Slough, UK). Lesion areas were determined by analyzing digital photographs using NIH Image J version 1.46r (Wayne Rasband, NIH) with calibration on a ruler included in each image. Lesion area data was subsequently analyzed using Prism (GraphPad Software, Inc., La Jolla CA). For mice challenged with the bioluminescent strain SAP149 only, in-life images of mice were obtained using a Xenogen Lumina II In Vivo Imaging System (IVIS) (PerkinElmer, Waltham MA) in the Veterinary Medicine Unit at the VA Ann Arbor Healthcare System. In-life imaging was performed at multiple time points according to the manufacturers protocols using Living Image 4.3.1 software (PerkinElmer) for instrument control, data acquisition, and analysis. Luminescence was determined within a standard circular region of interest (ROI) fully covering and individually centered on the challenge site of each mouse. Dark Background Subtraction, Flat Field Correction, and Cosmic Correction were used, with a Binning of 8 and no smoothing. Luminescence data was transferred to Microsoft Excel for formatting and subsequently to Prism for analysis. Images were captured with ROIs removed, with Color Scale Limits set to encompass the full range of intensities in all groups (25 to 6625) using a reverse rainbow Color Table, and with bioluminescence images superimposed on a white light photograph of the mice.

### Enzyme-linked immunosorbent assay

Antibody responses were assessed by ELISA essentially as described[45]. For analysis of antibodies specific for AT, wells of microtiter plates (Immulon 2, Thermo Labsystems, Franklin MA) were coated overnight at 4°C with 100 ng of recombinant alpha toxin (Product # H9395, Sigma Biochemicals, St. Louis, MO) in a 0.05 M carbonate buffer pH 9.5. Bound antibody was detected with secondary biotinylated antibody specific for rabbit IgG (Southern Biotechnology, Birmingham, AL) followed by streptavidin-alkaline phosphatase and 4-nitrophenylphosphate (Roche, Indianapolis, IN). Absorbance at 405 nm minus absorbance at 650 nm was determined using an ELISA reader (Emax microplate reader, Molecular Devices, Menlo Park, CA). Antibody titers were determined from serial two-fold dilutions of serum and represent the reciprocal dilution at the EC_50_ established using nonlinear regression to fit a variable slope sigmoidal equation to the serial dilution data using Prism 5.0 (GraphPad Software, Inc., San Diego, CA). Depending on assay setup, the lower limit of quantitation for the ELISA ranged from a reciprocal dilution of 16 to 4 as indicated in the figure legends. Commercial rabbit antisera against full length AT was employed as a positive control in the ELISA and TNA and was used in the repertoire analysis (Sigma, #S7531, Lot 042M4791: AT neutralization ED_50_ = 3,189; and Lot 112M4797, Sigma Biochemicals, St. Louis, MO) Each serum lot represents a pool comprised of sera from 2–3 hyperimmune rabbits. Serum from mice immunized with H35L was procured as described and was a generous gift of Dr. Drusilla Burns and Christopher Mocca (FDA)[54].

### Toxin Neutralization Assay

The ability of antibody to block AT cytotoxicity *in vitro* was assessed using the Jurkat T cell line (TIB-152, ATCC, Manassas, VA). Briefly, Jurkat T cells were grown in culture in RPMI with 10% fetal bovine serum, penicillin-streptomycin and 50 μM 2ME (complete medium) in a humidified 6.5% C0_2_ incubator. For each experiment, rabbit or mouse sera in duplicate was serially diluted with complete medium in polypropylene round-bottom 96 well plates in a final volume of 50 microliters per well. Recombinant AT reagent was prepared at four times (4X) the final concentration, with the final concentration (approximately 0.4 μg/ml) representing 3 to 4 multiples of the amount needed to kill 50% of the Jurkat T cells (Toxic dose 50% or TD_50_). Each TNA assay was validated by performance of a contemporaneous AT titration. Serially diluted rabbit or mouse antiserum was added to the AT and the mixture was returned to the incubator for 30 minutes, after which time, 100,000 Jurkat T cells in 0.1 ml of complete medium were added to each well. Following a 2-hour incubation, 20 microliters of WST-8 reagent (Genscript USA Inc., Piscataway, NJ) containing a water-soluble tetrazolium salt and electron mediator was added, and the absorbance at 450 nm minus absorbance at 650 nm was determined for each plate approximately 18 hours later using a Molecular Devices Emax microplate reader[55]. Neutralization ED_50_ (effective dilution at which 50% of cells are protected from cytotoxicity) titers were determined from serial two-fold dilutions of individual rabbit serum and represent the reciprocal dilution at the EC_50_ established using nonlinear regression to fit a variable slope sigmoidal equation to the serial dilution data using Prism 5.0 [56]. The TNA assay has a lower limit of quantification of 8. Samples with TNA titers below the lower limit of quantification were assigned a value of 8. For the analysis of peptide inhibition in the TNA, experimental serum samples were pre-incubated 1:1 with 32 μM of the 1–19 or 122–137 peptide for 1 hour at room temperature prior to analysis in the TNA.

### PND Ab binding studies

To assess whether PND-Ig inhibits the binding of AT to ADAM10-bearing cells, we employed an AT-erythrocyte ghost binding assay. Erythrocyte ghost receptors were prepared as described[57]. To evaluate whether PND-Ig inhibits binding of AT to red cell ghosts, 96 well polystyrene plates (Immulon 2) were coated with red cell ghosts diluted in a 0.05 M carbonate buffer pH 9.5 to an optical density at 600 nm of approximately 0.4 and incubated 2 hours at RT and then ON at 4C. For performance of the immunoassay, erythrocyte ghost-coated plates were blocked with 0.2 mls per well of 4% BSA in PBS for 1 hour at RT. Equal concentrations of rabbit PND-Ig and rabbit Control-Ig were mixed with 8 μg/ml (2X) of AT in PBS and incubated for 30 minutes at RT. Positive control antibodies were also mixed with identical amounts of AT, and included rabbit anti-AT (Sigma Biochemicals) and sheep affinity-purified anti-AT (SLHI101, Toxin technology, Sarasota FL) which were used at concentrations yielding neutralization equal to that of the rabbit PND-Ig. Following the 30 minute incubation with AT, experimental samples were applied to the plates and incubated 2 hours at RT. Plates were then washed three times and bound AT was then detected using a mouse mAb specific for the N-terminus of AT (8B7, IBT Bioservices, Gaithersburg MD) followed by a secondary biotinylated antibody specific for mouse IgG (Southern Biotechnology). Absorbance at 405 nm minus absorbance at 650 nm was determined using an ELISA reader. Results are presented as the percent of control AT binding determined through the use of the following formula: % Control AT binding = (OD_600_ with Ab/OD_600_ no Ab) x 100.

The ability of PND-Ig to inhibit oligomerization of AT was evaluated as described with some modification[37]. Briefly, 8 μg AT was pre-incubated with either 0.04 or 0.02 mls of PND-Ig, 0.04 mls of Control-Ig, or no Ab for 15 minutes at RT. The mixtures were then incubated on ice for 15 minutes with 0.5 mls of a 10% solution of rabbit RBCs in PBS (Innovative Research, Novi, MI). Samples were then spun at 14,000 RPM for 10 minutes and pellets were washed one time with PBS. RBCs were then lysed through incubation with 1.0 ml of red blood cell lysing buffer (Sigma Biochemicals) for 3 minutes. Red cell membranes were spun at 14,000 RPM for 10 minutes, resuspended in 0.09 ml of sample reducing buffer and 0.02 ml of each sample was loaded on a 4–15% gradient sodium dodecyl sulfate polyacrylamide gel for electrophoresis (BioRad, Carlesbad, CA). Following transfer of proteins to nitrocellulose by Western blot, membranes were probed with a 1:3000 dilution of sheep anti-AT Ab (SLHI101, Toxin technology) followed by an IRDye800-conjugated donkey anti-sheep Ab (Rockland Immunochemicals Inc., Limerick PA). Immunoflourescence was detected using an Odyssey imaging system (LI-COR Biosciences). AT heptamer was quantitated from greyscale non-compressed (tiff) images of the blot using NIH ImageJ software. For this, identically-sized boxes positioned to encompass the heptamer bands were created, nonsaturation of blacks was confirmed by inspection of the histogram, and integrated density was determined and data transferred to a spreadsheet for calculation.

### Statistical Analysis

For determination of ELISA EC_50_ and TNA ED_50_ titers, four parameter non-linear regression was used to fit variable slope sigmoidal equations to serial dilution data from the ELISA and TNA, respectively. Four parameter non-linear regression was also used to derive the PD_50_ metric using Ab dose and lesion area from the quantitative dermonecrosis titration data. The PA_50_ and PN_50_ determinations were derived from interpolations using the equations obtained from application of linear regression to the serum Ab and TNA titers obtained from the quantitative dermonecrosis titration. For lesion analysis and assessment of bacterial burden, the non-parametric Mann-Whitney U and Kruskal-Wallis test with Dunn’s multiple comparison post test were used to assess significance between two or more than two groups, respectively. For analysis of rabbit TNA data, mouse and rabbit Ab data, and the AT-erythrocyte ghosts binding assay data, one way ANOVA with Tukey’s multiple comparison post test was used for comparisons among more than two groups. For all statistical analysis, a *p* value of < 0.05 was considered significant. All statistical analysis was performed using GraphPad Prism software version 5 (GraphPad Software, Inc., San Diego, CA).

## Results

### A neutralizing epitope exists within the pore-forming domain of AT

**Fig 1:**
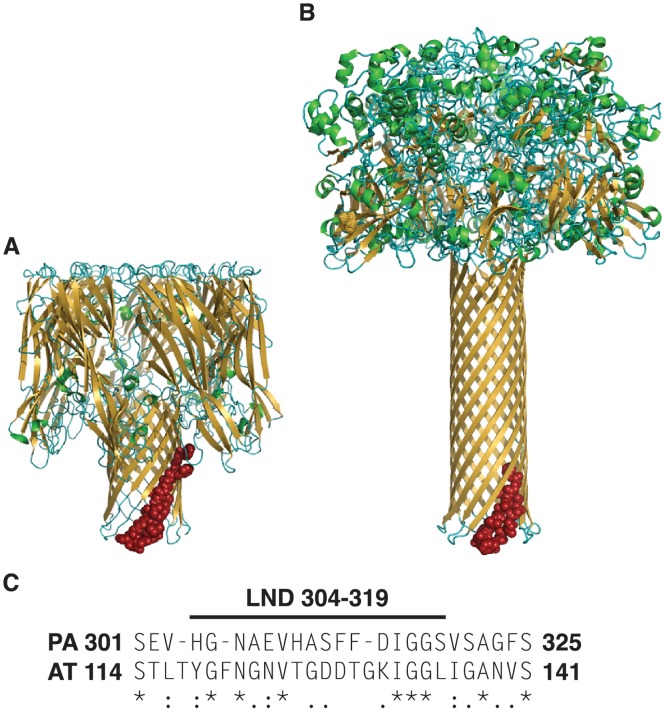
Alpha toxin and protective antigen share structural and functional homology but only limited sequence identity. Comparison of protein structural models of the heptameric AT (PDB7AHL) (A) and PA (PDB1V36) (B). Sequences in red represent aligned sequence shown in (C). Amino acid alignment of PA and AT in the region of the LND of PA demonstrates 37% sequence identity. Asterisks denote positions of amino acid identity, while periods and colons denote semi-conservative and conservative substitutions, respectively.

**Fig 2:**
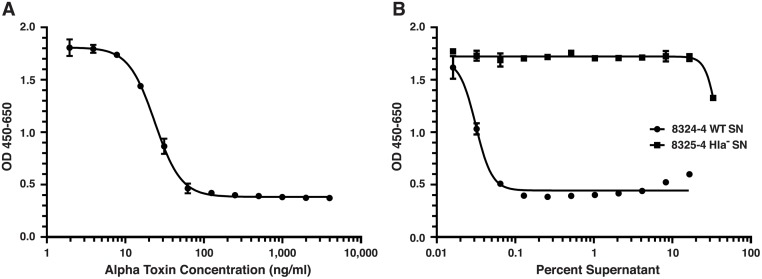
Jurkat T cells are sensitive to recombinant and native alpha toxin in vitro. Shown are representative titrations demonstrating the cytotoxicity on Jurkat T cells in vitro of recombinant alpha toxin (A) and native alpha toxin (B) from the supernatants of overnight cultures of *S*. *aureus* 8325–4 (circles) or the isogenic (*hla*-) deletion mutant of 8325–4 (squares). Optical density (OD) increases with viability. The EC_50_ for recombinant AT = 23.75 ng/ml and for native AT from the supernatant of the 8325–4 strain = 0.03%. The supernatant from *S*. *aureus* 8325–4 (*hla*-) was not cytotoxic (B).

The structural and functional similarities of AT and PA led us to hypothesize that a linear neutralizing epitope exists in the pore-forming domain of AT. To test this hypothesis, we performed an initial experiment to elicit Ab in rabbits to a.a. 122–137 in AT. This region in AT demonstrates some structural and functional homology with the 22–23 loop of PA, but only 37% sequence identity when aligned (Fig. 1). We synthesized a MAP displaying the a.a sequence 122–137 from AT positioned at the C-terminus of the T* helper T cell epitope from the circumsporozoite protein of *Plasmodium falciparum*. We and others have successfully incorporated this MHC Class II-restricted helper T cell epitope into MAPs where it provides cognate help towards covalently-linked B cell epitopes [44,48,49]. In order to assess the in vitro functional activity of antisera raised to the initial MAP construct, and to other constructs employed in these studies, we required a robust, sensitive and reproducible toxin neutralization assay. While prevention of rabbit erythrocyte lysis has been used to evaluate the neutralizing potential of antisera against AT in the *S*. *aureus* model, we sought an in vitro assay based on a cell type which was likely a pathophysiologically important immune cell target for AT in vivo. Both mouse and human T cells are highly sensitive to the effects of AT in vitro, and along with other leukocytes, are susceptible and pathophysiologically-relevant cell targets for AT in vivo [24,58,59]. We, therefore, developed an optimized toxin neutralization assay (TNA) which utilizes Jurkat, an immortalized human T cell line, which we found to be highly and reproducibly sensitive to recombinant AT, and to native AT from culture supernatants of MRSA (not shown) and MSSA strains (Fig. 2A, 2B).

**Fig 3:**
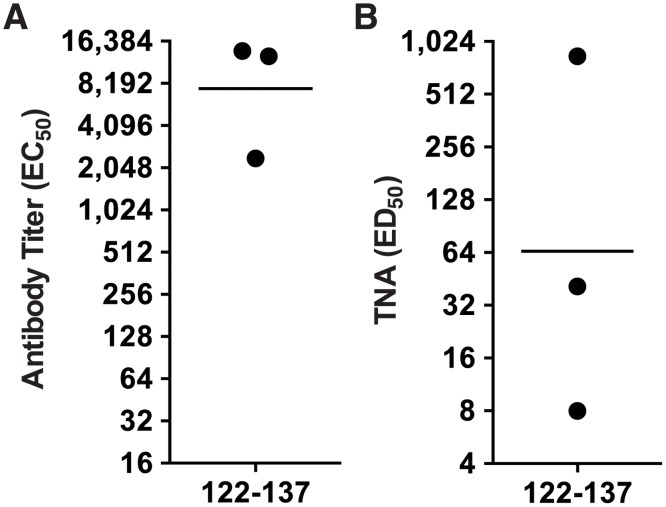
Antibody against a.a. 122–137 binds AT by ELISA and protects Jurkat T cells in vitro. Three rabbits were immunized four times with a MAP displaying a.a. 122–137 from AT in an emulsion with CFA for priming immunizations and IFA for booster immunizations. Approximately 10 days after the final immunization, rabbit were bled and sera was evaluated by ELISA for reactivity with full length AT (A) and in the TNA (B).

**Fig 4:**
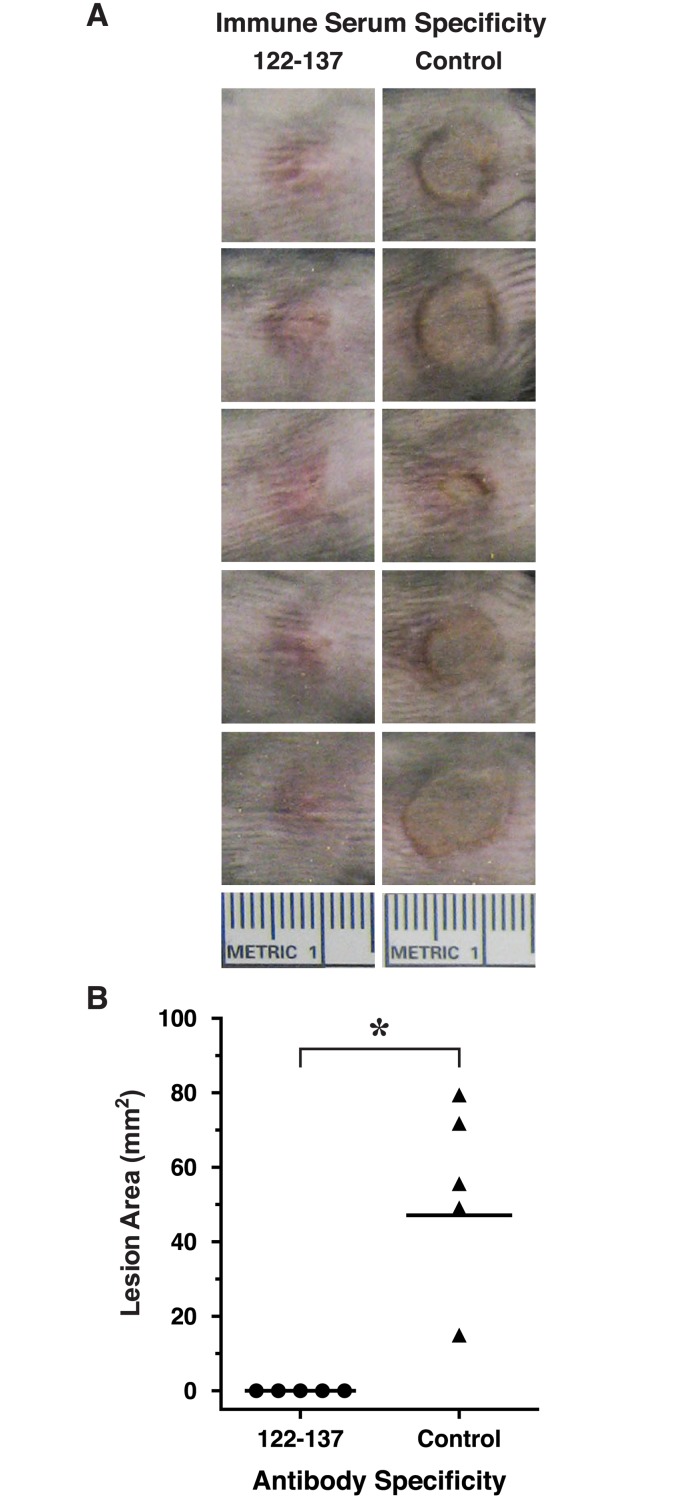
Passive immunization of mice with AT-neutralizing antisera specific for a.a. 122–137 protects mice from dermonecrosis. Five C57BL/6 mice/group were passive immunized i.p. with 0.5 mls of rabbit serum specific for a.a. 122–137 of AT, or irrelevant control rabbit serum. One day later, all mice were challenged i.d. with 20 x 10^6^ CFUs of *S*. *aureus* 8325–4 and were followed for 48 hours. After 48 hours, all mice were euthanized and dermonecrotic lesion areas were photographed (A) and lesion areas were determined as described in *Material and Methods* and displayed graphically (B). Horizontal bars are geometric means. * *p* = 0.0037, Mann-Whitney U test, one-tailed.

Female New Zealand White rabbits (n = 3) were immunized 4 times at two-week intervals with the MAP 122–137 and serum was collected two weeks after the 4^th^ immunization for analysis. Sera from all three rabbits immunized with MAP 122–137 were found to be immunoreactive with immobilized AT by ELISA (Fig. 3A). When analyzed in the TNA, serum from one of the rabbits immunized with the MAP 122–137 demonstrated significant neutralization of AT in vitro (Fig. 3B). To evaluate whether this neutralizing activity would translate into mitigation of dermonecrosis following *S*. *aureus* infection, we passively immunized groups of C57BL/6 mice (n = 5) i.p. with the neutralizing serum from the MAP 122–137-immunized rabbit, or with serum from a rabbit immunized with a T*-containing LND MAP immunogen (negative control)[44]. One day later, mice were challenged intradermally (i.d.) with 20 x 10^6^ CFUs of *S*. *aureus* 8325–4, an MSSA strain which expresses high levels of AT[60,61]. Two days after bacterial challenge, mice were euthanized, photographed and lesion areas were determined. Mice passively immunized with the 122–137-specific rabbit antisera were completely protected from dermonecrosis, while mice passively immunized with the negative control antisera developed significant dermonecrosis (*p* = 0.0037, Fig. 4). The results confirmed that a neutralizing epitope, which we refer to as the pore neutralizing determinant (PND), is present in the β-pore forming domain of AT.

### In vivo mapping of the pore neutralizing determinant in AT

**Fig 5:**
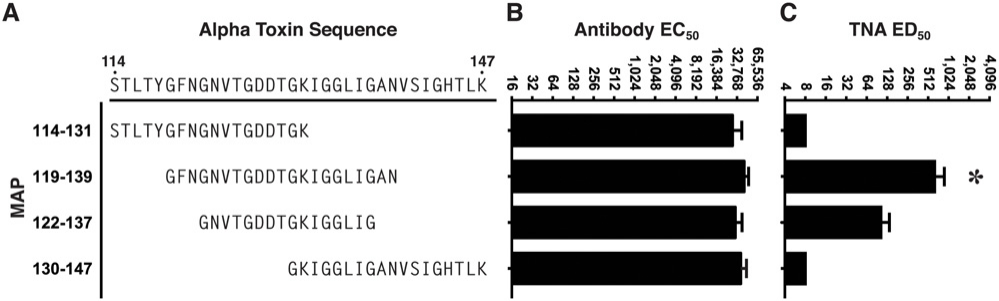
Antibody and TNA responses from rabbits immunized with MAP constructs displaying overlapping residues from AT. Groups of rabbits were immunized 4 times at two-week intervals with a mixture of two MAPs each containing the respective B cell target sequence (A) linked separately to the T* and P30 helper T cell epitopes. Ten days after the final immunization, rabbits were bled and sera were evaluated by ELISA for reactivity with immobilized full length AT (B) or the in the TNA (C). Bar charts represent arithmetic means. Error bars represent SEM. * *p* = 0.022, one-way ANOVA; *p*< 0.05, Tukey’s multiple comparison test: 119–139 vs all other groups.

**Fig 6:**
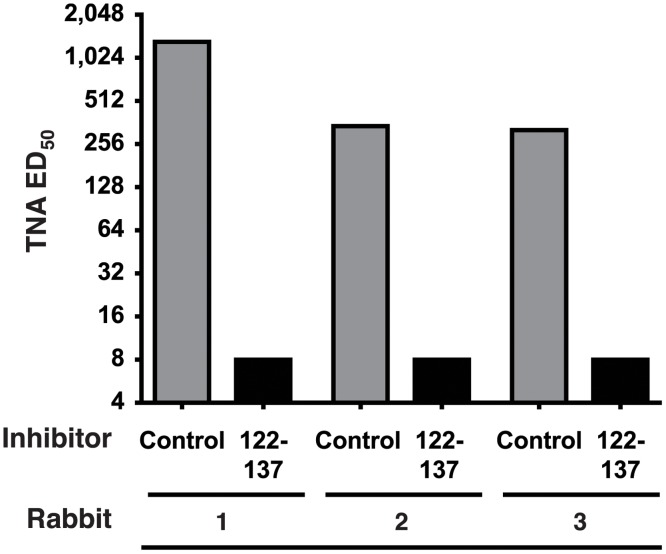
The 122–137 peptide inhibits AT neutralization activity in the sera of rabbits immunized with MAP-119–139. Antiserum from each of 3 rabbits immunized four times with the MAP-119–139 shown in Fig. 5, was preincubated with 32 μM of the linear peptide, a.a. 122–137 or a control linear peptide for 1 hour at RT prior to assessment in the TNA.

To more precisely map the PND of AT in vivo, we designed and synthesized MAPs for eliciting antibody against 3 additional overlapping sequences from AT, in addition to the 122–137 sequence (Fig. 5A). As in the first experiment, each MAP was synthesized collinearly with the T* helper T cell epitope, but in addition, all 4 MAPs were also separately synthesized with replacement of the collinear T* with the P30 helper T cell epitope from tetanus toxin, which is also an effective source of linked helper T cell stimulation in outbred rabbits [43,50]. Groups of rabbits (n = 3), in turn, were immunized with a mixture of two MAPs, each containing the identical B cell target sequence from the β-pore domain of AT, but with separate, independent helper T cell epitopes, an approach we have previously determined increases antibody responses and decreases non-responsiveness in outbred rabbits. As shown in Fig. 5B, all 4 MAP constructs elicited high-titer Ab reactive with AT by ELISA. When analyzed in the TNA, antisera from rabbits immunized with the 119–139 sequence demonstrated significantly higher levels of neutralization when compared to the antisera from rabbits immunized with the 122–137 sequence, or to antisera from rabbits immunized with MAPs displaying sequences N- and C-terminal to the 122–137 sequence, which had no detectable neutralization (*p* = 0.022, Fig. 5C). The neutralizing antibody in the sera from all three rabbits immunized with the MAP 119–139 was, however, completely inhibitable in vitro by incubation with a 122–137 linear peptide prior to analysis in the TNA (Fig. 6). These data confirm that the PND neutralizing epitope is linear in nature, and suggest that though the 21-residue 119–139 segment is more effective at eliciting the neutralizing antibody specificities, the critical residues defining the PND appear to fall within the nested 16-residue 122–137 segment.

### PND-specific antibody prevents *S*. *aureus*-induced dermonecrosis in vivo

**Fig 7:**
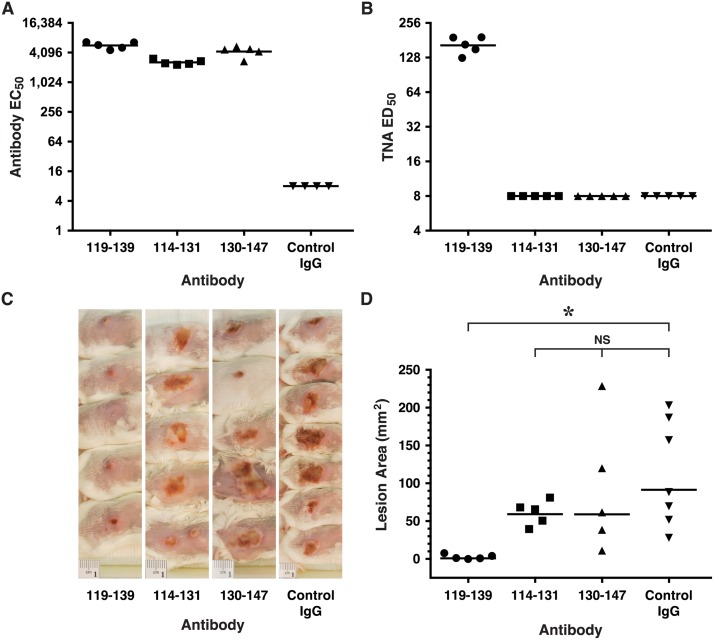
Rabbit-IgG specific for the 119–139 sequence from AT protects mice from LAC/USA300-mediated dermonecrosis. Groups of BALB/c mice (n = 5 except control IgG, n = 7) were passively immunized s.c. with normalized volumes of the respective affinity purified rabbit sera to establish approximately equivalent serum antibody titers in recipient mice. Approximately 48 hours later, all mice were bled and individual mouse sera were evaluated by ELISA for reactivity with immobilized AT (A) and in the TNA (B). All groups were then challenged i.d. with 38 x 10^6^ LAC/USA300. Forty-eight hours later, all mice were euthanized, lesions were photographed (C) and lesion areas determined (D). Mice passively immunized with rabbit IgG specific for the 119–139 sequence had significantly smaller lesions than mice receiving Control IgG (*p = 0.008, Kruskal Wallis; *p*< 0.05, Dunn’s multiple comparison test: 119–139 vs Control IgG). Lesion areas from groups of mice receiving the 114–131 and 130–147 IgG were not significantly different from the lesion areas of mice receiving the irrelevant Control-IgG, nor were they significantly different from the lesion sizes of mice administered 119–139 IgG, as assessed using the Dunn’s post-hoc analysis. Horizontal lines in (A), (B) and (D) represent geometric means.

To assess whether the TNA results would predict in vivo efficacy, we purified IgG from the sera of each rabbit with the highest Ab titer among each group of rabbits immunized with either the 119–139, 114–131 and 130–147 sequences, and then passively immunized groups of BALB/c mice (n = 5) s.c. with the respective rabbit IgG. Two days later, mice were bled and then challenged i.d. with 38 x 10^6^ LAC/USA300. We employed LAC/USA300 for these challenges since this MRSA strain is known to produce high levels of AT [35], is highly virulent in the dermonecrosis model, and is the predominant strain implicated in CA-MRSA infections in the community [27,35]. Analysis of mouse sera obtained on the day of challenge revealed that all groups of mice had approximately equivalent levels of serum Ab by ELISA, but as expected, only sera from mice passively immunized with the 119–139- specific IgG demonstrated neutralization in the TNA (Fig. 7A,7B). Two days after challenge, all mice were sacrificed and dermonecrotic lesions were photographed and analyzed. Mice passively immunized with 119–139-specific IgG were significantly protected from dermonecrotic lesions compared to mice passively treated with the Control-IgG (*p* = 0.008, Fig. 7C, 7D). There were no statistical differences among lesion size in groups of mice passively immunized with the 114–131, 130–147 and Control-Ig.

### Antibody specific for the PND is highly potent for preventing dermonecrosis

**Fig 8:**
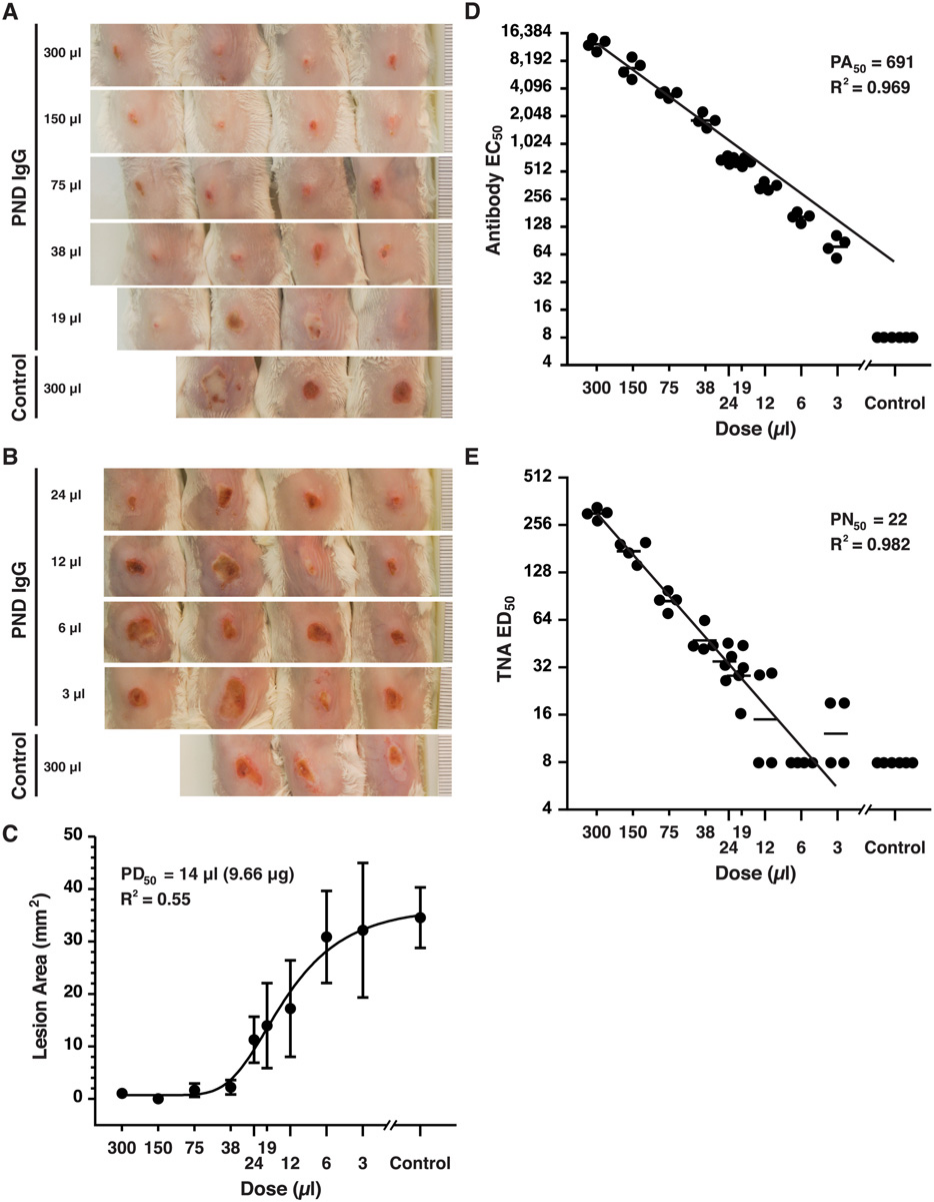
Dose-Response Titration of PND Ab for protection of mice from LAC/USA300 Dermonecrosis. Groups of BALB/c mice (n = 4 except neg. controls, n = 3) were passively immunized s.c. with two-fold dilutions of PND Ab starting with a dose of 300 μl (panel A) or 24 μl (Panel B). 48 hours later, mice were challenged i.d. with 32 x 10^6^ (panel A) or 28 x 10^6^ (panel B) CFUs LAC/USA300. Two days later, all mice were euthanized and photographed and lesion areas determined (C). Group-specific lesion area data were plotted against dose and four parameter non-linear regression was used to determine the EC_50_ of 14 μl (9.66 μg) (R^2^ = 0.55) which represents the dose of PND-Ig which prevented 50% of the maximal lesion area. Antibody and neutralization titers from the sera of mice passively immunized with PND-Ig were determined by ELISA (D) and in the TNA (E) from serum obtained from individual mice on the day of challenge. Each circle represents an individual mouse data point and horizontal lines represent geometric means. Regression lines derived from the serum antibody and TNA data vs. dose were then used to interpolate the PA_50_ and PN_50_ which are expressed as reciprocal titers. The regression equation for PA_50_ determination was log ELISA EC_50_ = 0.9504 x log Dose + 1.75, and for PN_50_ determination was log TNA ED_50_ = 0.858 x log Dose + 0.3589.

Work in the anthrax model has shown that the LND is a highly sensitive target for humoral immunity, as low levels of antibody against these sequences are capable of neutralization in vitro and mediation of protection in vivo in the rabbit inhalation anthrax challenge model [42,44]. In a series of two experiments, we assessed whether this was true of the PND as well through quantitative determination of the potency of PND-Ig in vivo for passive protection of mice from LAC/USA300-mediated dermonecrosis. Additionally, we desired to establish the corresponding in vitro surrogates, namely antibody and neutralization titers, that might be used to quantitatively predict protection from dermonecrosis in this model. In the first experiment, groups of BALB/c mice (n = 4) were passively immunized s.c. with serial two-fold dilutions of PND-Ig beginning at a dose of 300 μl per mouse or 0.2 mg of PND-specific Ab. Two days later, mice were challenged intradermally with 32 x 10^6^ CFUs of LAC/USA300. As shown in Fig. 8A, all doses between 300 μl to 38 μl almost completely prevented lesion formation compared to the negative control group. A follow-on titration in age-matched mice was then performed to extend the titration to lower doses, beginning with a top dose of 24μl (Fig. 8B). As in the first titration, mice were challenge 48 hours after passive immunization with LAC/USA300 and in this challenge received 28 x 10^6^ CFUs. Two days after bacterial challenge, all mice were sacrificed and photographed (Fig. 8B). The procedures and actual challenge doses for the two experiments were nearly identical, and the treatment doses overlapped, enabling the results from the two titrations to be combined and analyzed collectively. Four parameter non-linear regression was used to derive the dose of PND-Ig required to prevent 50% of lesion size (Protective Dose 50% or PD_50_) which was 14 μl or 9.66 μg (R^2^ = 0.55) in these experiments (Fig. 8C). To derive the in vitro correlates for protection from dermonecrosis, individual mouse sera obtained prior to challenge were analyzed in the ELISA and TNA. The reciprocal serum antibody (Protective Antibody 50%, PA_50_) and neutralization (Protective Neutralization 50%, PN_50_) titers associated with prevention of 50% of maximal lesion formation in the dermonecrosis model were then interpolated from regression lines derived from the X-Y plots of the ELISA and TNA data vs. dose (Fig. 8D, 8E). The PA_50_ and PN50 expressed as reciprocal antibody titer and neutralization titers were 691 (R^2^ = 0.969) and 22 (R^2^ = 0.969), respectively.

### Antibody specific for the PND enables host control of bacterial growth in vivo

**Fig 9:**
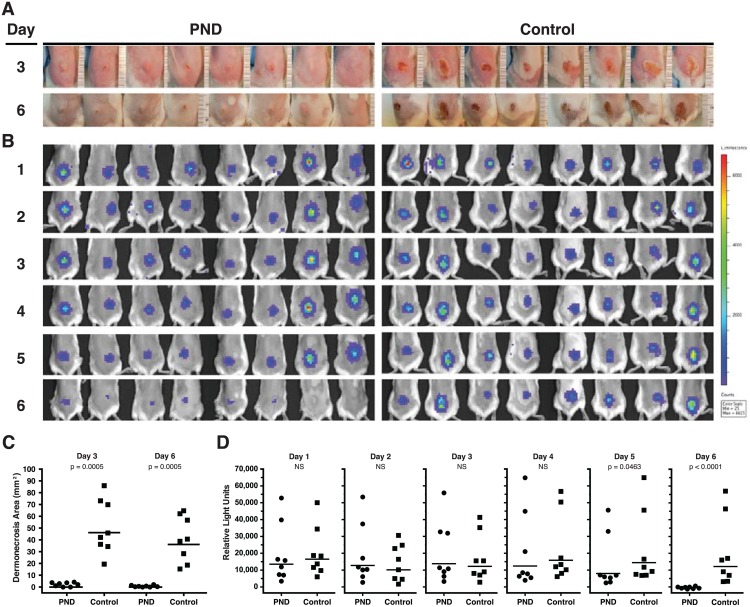
Passive immunization with PND-Ig suppresses bacterial growth in vivo. Groups of mice (n = 8) were passively immunized with 0.3 mls of PND-Ig or Control Ig 48 hours prior to i.d. challenge with 40 x 10^6^ SAP149, a luminescent derivative of the USA300 stain NRS384 [53]. (A) All mice were photographed at 3 and 6 days after challenge for determination of lesion size as described in *Material and Methods*. (B) All mice underwent daily in-life imaging for detection of bioluminescence beginning one day after challenge (day 1) as described in *Material and Methods*. (C) PND-Ig mice were significantly protected from dermonecrosis compared to control-Ig mice at day 3 and day 6 (*p* = 0.0005, day 3; *p* = 0.0005, day 6, Mann-Whitney U, one-tailed). (D) PND-Ig mice had significantly less luminescence and bacterial burden at days 5 and 6 after challenge compared to Control-Ig mice (*p* = 0.046, day 5; *p*< 0.0001, day 6, Mann-Whitney U, one-tailed).

To establish whether Ab specific for the PND can decrease bacterial burden over time in mice challenged with USA300, we employed passive immunization with PND-Ig, followed by challenge with SAP149, a luminescent and virulent derivative of the USA300 MRSA strain NRS384, which enabled in-life serial monitoring of bioluminescence as a validated surrogate for ex vivo CFU determination [24,32,53,62]. Prior to use of this strain for challenge, we determined that SAP149 produces high levels of AT, though approximately 25% lower than the amounts produced from LAC/USA300 evaluated in contemporaneous titrations of culture supernatants in the TNA (not shown). As a consequence, we administered challenge doses approximately 25% higher for SAP149 compared to prior experiments with LAC/USA300. Groups of BALB/c mice (n = 8) were passively immunized s.c. with 0.3 mls of PND-Ig or control affinity purified Ab 48 hours prior to i.d. challenge of individual mice with 40 x 10^6^ CFUs of SAP149. Mice were then serially imaged by IVIS for bioluminescence every 24 hours for 6 consecutive days. As expected, mice administered PND-Ig had significantly smaller lesions than mice treated with control-Ig when lesion areas were determined on days 3 and 6 (*p* = 0.0005, day 3; *p* = 0.0005, day 6, Fig. 9A, 9C). Despite the highly significant mitigation of dermonecrosis, however, the bacterial burdens of the PND-Ig and Control-Ig treated mice were not statistically different until days 5 and 6, whereupon the PND-Ig group demonstrated significant reductions in bacterial burden compared to the Control-Ig group of mice (*p* = 0.046, day 5; *p*< 0.0001, day 6, Fig. 9B, 9D). Indeed, on day 6, bioluminescence in the PND-Ig-treated mice had approached background levels, whereas the day 6 bioluminescence in the Control-Ig treated mice was 97% of their Day 1 bioluminescence levels (Fig. 9D). The reductions in dermonecrotic lesion size and ultimately, in bacterial burden in the mice treated with PND-Ig were associated with serum geometric mean ED_50_ reciprocal TNA titers of 246 and 96 on the day of challenge (Day 0) and on Day 6, respectively (not shown).

### Antibody specific for the PND does not block AT binding to cellular receptors but does inhibit heptamerization of AT

**Fig 10:**
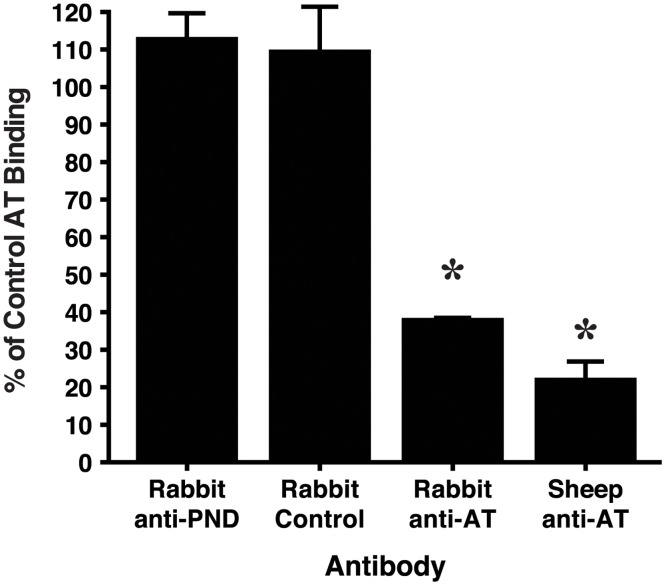
PND-specific Ab does not block binding of AT to RBC ghosts. RBC ghosts were immobilized on polystyrene 96 well plates and were blocked with 4% BSA in PBS. Soluble AT was incubated at RT with AT alone, or with AT mixed with identical concentrations of either PND-Ig or Control-Ig, or with rabbit anti-AT or sheep anti-AT at concentrations mediating neutralization equal to that of the PND-Ig. Binding of AT was detected using a mouse mAb specific for the N-terminus of AT as described in methods. *p< 0.0001 by ANOVA; *p*< 0.05, Tukey’s multiple comparisons test: Rabbit anti-AT and Sheep anti-AT vs both Rabbit anti-PND and Rabbit Control. Bars represent arithmetic means from quadruplicate samples and error bars indicate +/- 1 SEM. % Control AT binding = (OD_600_ with Ab/OD_600_ no Ab) x 100. The results are representative of 2 separate assays.

We next endeavored to determine the mechanism by which PND-specific Ab neutralizes AT in vitro and mitigates dermonecrosis in vivo. First, we evaluated whether PND-Ig inhibits the binding of soluble AT to ADAM10 through use of immobilized RBC ghosts according to a previously described method[57]. As shown in Fig. 10, neither the PND-Ig nor Control-Ig inhibited the binding of AT to the RBC ghosts, whereas both polyclonal rabbit and sheep Ab specific for AT demonstrated significant inhibition of AT binding (*p*< 0.0001).

**Fig 11:**
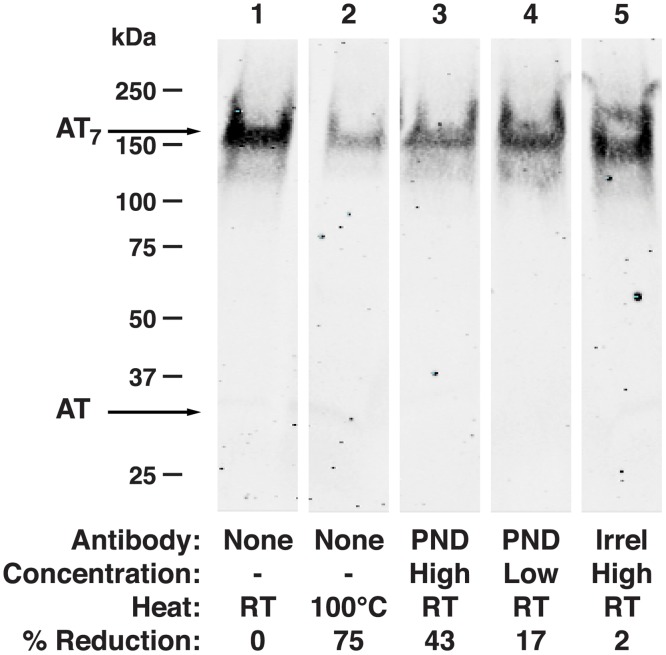
PND-specific Ab inhibits heptamerization of monomeric AT. Rabbit RBCs were incubated with AT or with AT pre-incubated with PND-Ig or Control-Ig as described in methods. Following RBC lysis, red cell membranes were pelleted and washed, resuspended in sample reducing buffer and run on 4–15% gradient gels by SDS-PAGE. Excepting the sample in lane 2, samples were not boiled or otherwise heated prior to electrophoresis. AT bands were detected with sheep anti-AT Ab followed by IRDye800-conjugated donkey anti-sheep IgG, and immunoflourescence was detected using an Odyssey imaging system. Image density was quantified using NIH ImageJ software. Results are representative of 3 separate assays.

Prior studies have shown that neutralizing Abs specific for the N-terminus of AT act through inhibition of heptamerization, which is a required step for AT-mediated pore-formation and cell lysis [36,37,63]. Since the N-terminus and the glycine-rich loop comprising the PND may interact in the formation of the lytic pore[64], we evaluated whether PND-specific Ab, like Ab to the N-terminus of AT, acts by inhibiting heptamer formation. Employing a previously described assay using RBCs, PND-Ig but not Control-Ig was found to inhibit heptamerization (Fig. 11).

### Antibody specific for the PND is a minor component of the Ab repertoire elicited to full length AT in rabbits and mice

**Fig 12:**
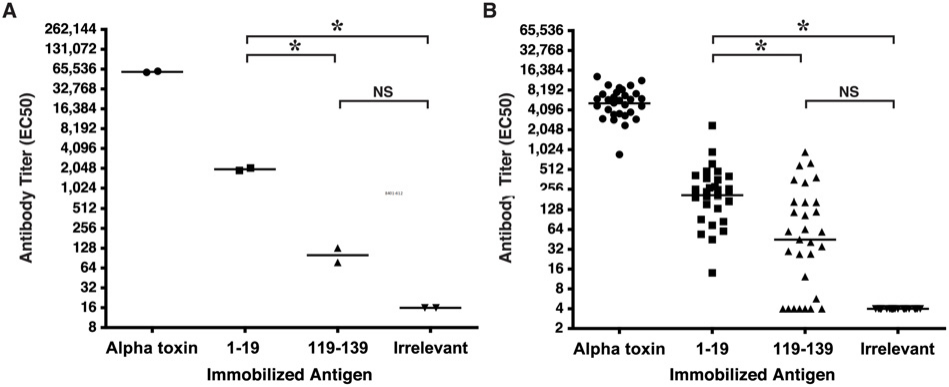
PND-specific Ab is a minor component of the repertoire of Abs elicited following AT immunization of rabbits and mice. (A) Two independent lots of commercially available antisera each representing pooled antisera from 2–3 rabbits immunized with full length AT, were evaluated for reactivity with immobilized AT or with peptides by ELISA. The geometric mean titer for antibody reactive with the 1–19 peptide sequence was significantly higher than the titers of Ab reactive with the 119–139 or irrelevant peptide sequences (*p = 0.0002, One way ANOVA; Tukey’s multiple comparison test: *p<0.05, 1–19 vs. 119–139 and 1–19 vs Irrelevant). The titers of Ab reactive with the 119–139 and Irrelevant peptide sequences were not statistically different (B) Individual mouse antiserum obtained from mice immunized with H35L were evaluated for reactivity with immobilized AT or with peptides by ELISA. Geometric mean antibody titers specific for the 1–19 peptide sequence was significantly higher than the responses to either the 119–139 or irrelevant peptide sequences (*p* = 0.0001, One way ANOVA; Tukey’s multiple comparison test: *p<0.05, 1–19 vs. 119–139 and 1–19 vs Irrelevant). The titers of Ab reactive with the 119–139 and irrelevant peptide sequences were not statistically different. Each immobilized MAP was validated with a control antiserum (not shown). Bars represent geometric means. The lower limit of quantitation for analysis of rabbit and mouse antibody titers were 16 and 4, respectively.

One distinctive attribute of the LND from PA is the finding that antibody to this epitope is not significantly represented among the repertoire of Abs elicited when rabbits, non-human primates or humans are immunized with vaccines containing full length PA. In effect, the LND epitope is immunosilent in whole PA. To evaluate whether the PND by analogy might be similarly immunosilent in AT, we evaluated two separate commercially available lots of high-titer, rabbit antisera against full length AT for the presence of antibody immunoreactive with the 119–139 peptide, which contains the minimal PND neutralizing determinant, a.a. 122–137, defined in these studies. Each lot represents a pool from 2–3 rabbits immunized with AT. Though both lots were determined to have high-titer Ab against AT (reciprocal geometric mean EC_50_ = 69,723), neither pool had greater than background reactivity with the immobilized 119–139 peptide. For comparative purposes, both pools were found to have significant Ab titers against a control peptide comprised of the N-terminal 1–19 amino acids from AT which we have separately determined contains a relatively immunodominant neutralizing epitope (*p* = 0.0002, Fig. 12A)[65]. A similar analysis was performed on individual sera obtained from mice immunized with H35L from a separate independent study[54]. Though serum samples from all mice (n = 30) were found to have good Ab responses against full length AT (reciprocal geometric mean EC_50_ = 5,172), the serum Ab titers immunoreactive with the 119–139 peptide were not statistically different from the titers reactive with the irrelevant peptide control (Fig. 12B). Some individual mice, however, did have meaningful levels of Ab reactive with the 119–139 peptide. Like the rabbit antisera, the mouse antisera did have significant Ab titers against the N-terminal 1–19 peptide sequence, compared to the Ab titers reactive with the 119–139 and Irrelevant peptide sequences (*p* = 0.0001).

## Discussion

Like PA from *B*. *anthracis*, AT is a member of a class of β-barrel pore forming toxins, a group which also includes the leukotoxins of *S*. *aureus*, but also members that multimerize to form larger pores, such as streptolysin O, aerolysin, *Clostridium perfringens* perfringolysin O, among other toxins [40](reviewed in [66]). These toxins all employ a β-loop structure to cross the membrane in the assembled pore. The structural and functional similarities of AT with PA led us to hypothesize that AT, like PA, might contain an important neutralizing epitope in the region of AT analogous to the 2β2–2β3 loop of PA where the LND is found. The linear nature of the LND, and the potency of antibodies against this site for in vitro toxin neutralization and ultimately, for protection of rabbits from lethal aerosol spore challenge with *B*. *anthracis*, are qualities that would be highly desirable for a neutralizing epitope against AT for inclusion in a mono- or multivalent epitope-focused vaccine against *S*. *aureus*[42–45]. Our data demonstrates that a linear neutralizing epitope, which we refer to as the pore neutralizing determinant (PND), is present in the β-pore region of AT, likely within residues 122–137. Rabbit antibody elicited to a MAP displaying a.a. 119–139 from AT, but not to adjacent sequences within the β-pore region, elicited antibody capable of neutralization in vitro, and the antibody-mediated neutralization was completely inhibited by a linear peptide comprising a.a. 122–137. The linear nature of this neutralizing epitope, as opposed to a conformational epitope, comprised of non-contiguous sequences, markedly increases the likelihood that this sequence could be successfully exploited in the development of a mono- or multivalent vaccine for *S*. *aureus*. As demonstrated with the LND, such linear epitopes retain their immunogenicity and utility in varied immunogen forms including simple peptides, MAPs and recombinant proteins, and would likely be immunogenic and elicit neutralizing antibody in other immune formulations as well, including virus-like particles and recombinant constructs expressed in eukaryotic expression vectors [42–45].

Like antibody specific for the LND, antibody against the PND appears to be potent. Employing a quantitative LAC/USA300 dermonecrosis model, the PND-Ig was found to have a PD_50_ of 0.014 mls or 9.66 μg. The potency determinations are only relative, however, as it was out of scope in the current study to compare the potency of Ab to the PND to other epitope-specific immunotherapeutics or monoclonal Abs capable of neutralizing AT. Such quantitative comparisons in vivo, using this or other quantitative in vivo models, could prove highly informative for reproducibly characterizing and contrasting the potency of immunotherapeutics in the dermonecrosis model, and in other systemic models of *S*. *aureus* infection.

The potency of PND-Ig may also be reflected in the levels of serum neutralization (PN_50_) associated with the PD_50_. Serum neutralization established using the human T cell-based TNA in the current study appears to be a reliable in vitro correlate for protection in the dermonecrosis model. Unlike anthrax, where the presence of a single bi-component virulence factor comprised of lethal factor and protective antigen, has facilitated and enabled development of a highly predictive and useful in vitro surrogate of protective immunity [56,67], no single enabling assay has emerged for *S*. *aureus*. Recent data from mouse models, however, combined with comparative analyses of the virulence factors likely responsible for the increased virulence of USA300 strains in the community, increasingly suggest that AT may play a more singularly critical role in the pathogenesis of *S*. *aureus* infections, especially with regard to tissue injury, inflammation, and immune evasion, than may have previously been appreciated. This may emphasize the utility of a more universally adopted and standardized TNA for the evaluation of vaccines and immunotherapeutics against AT[18,19,25,26]. The Jurkat T cell line is highly and reproducibly sensitive to AT, is easily maintained in culture, and T cells have been shown to be a highly critical participant in the innate and adaptive immune response to *S*. *aureus* infection. In particular, γδ T cells play a prominent role in initiating a cascade of events which lead to the recruitment of neutrophils and other effectors to the site of *S*. *aureus* infection, and in their absence, intradermal challenge of mice with *S*. *aureus* results in significantly larger cutaneous lesion development and substantially higher lesional bacterial counts compared to mice with normal populations of γδ T cells [24,59].

Protection of T cells and other immune effector cells from the toxic effects of AT following intradermal challenge with the USA300 is one possible mechanism explaining why Ab against the PND is capable of mediating control of bacterial proliferation in the current study. While mice treated with Ab against the PND were significantly protected from dermonecrosis when lesion areas were assessed at day 3, mitigation of bacterial proliferation was marginally significant at day 5 and was highly significant by day 6. This juxtaposition of significant mitigation of dermonecrosis with no detectable effect on bacterial burden in the early stages of cutaneous infection has been noted in other studies in this model [22], and suggests, as has been noted in the cutaneous and pneumonia mouse models, that AT binding to ADAM10 rapidly disrupts epithelial barriers in a manner that is independent of bacterial proliferation. In support of this interpretation, elegant studies have demonstrated that mice with a conditional knockout of ADAM10 in the skin, or in the respiratory epithelium, are resistant to *S*.*aureus* mediated dermonecrosis and lethal pneumonia, respectively, yet in both instances, the conditional ADAM10 knockout mice had bacterial burdens which were not statistically different from the wild type mice [68,69]. The delay in the observed effect of the PND-Ig to control bacterial burden may be explained by the use of very high challenge doses (40 x 10^6^ CFUs/mouse) of *S*. *aureus*, which may have overwhelmed the capacity of the neutralizing Ab to oppose the effects of AT in facilitating bacterial growth.

Finally, though immunization of mice and rabbits with H35L and other full length forms of AT reproducibly elicit neutralizing Ab, few neutralizing epitopes in AT have been described to date. Monoclonal Abs that neutralize AT bind conformational epitopes in the cap region of AT in one case, and to sequences yet to be fully elucidated [36,57,70]. Two independent studies have demonstrated that at least one or more neutralizing epitopes may exist in the N-terminal 62 amino acids of AT, though it is currently unclear whether these epitopes are linear or conformational in nature [36,37]. We recently determined that a relatively immunodominant linear neutralizing epitope exists within a.a. 1–19 at the N-terminus of AT[65]. In contrast to this immunodominant N-terminal epitope, the data in the current study suggest that the PND may be relatively immunorecessive in rabbits, and perhaps less so in mice, though definitive characterizations of the immunogenicity of this region in the β-pore of AT will require more definitive studies in larger number of animals[71,72]. Is is also not known whether immunization of humans with H35L or other full length forms of AT would elicit Ab specific for the PND, or alternatively, that generation of significant levels of antibody to this neutralizing epitope in the β-pore region of AT will require immunization with an epitope-focused, non-native immunogen.
